# New Application of Psoralen and Angelicin on Periodontitis With Anti-bacterial, Anti-inflammatory, and Osteogenesis Effects

**DOI:** 10.3389/fcimb.2018.00178

**Published:** 2018-06-05

**Authors:** Xiaotian Li, Chunbo Yu, Yi Hu, Xinyi Xia, Yue Liao, Jing Zhang, Huiwen Chen, Weili Lu, Wei Zhou, Zhongchen Song

**Affiliations:** ^1^Department of Periodontology, Ninth People's Hospital, Shanghai Jiao Tong University School of Medicine, Shanghai, China; ^2^Shanghai Key Laboratory of Stomatology & Shanghai Research Institute of Stomatology, National Clinical Research Center of Stomatology, Shanghai, China; ^3^Ninth People's Hospital, Shanghai Jiao Tong University School of Medicine, Shanghai, China; ^4^Laboratory of Oral Microbiota and Systemic Disease, Shanghai Research Institute of Stomatology, Ninth People's Hospital, Shanghai Jiao Tong University School of Medicine, Shanghai, China

**Keywords:** psoralen, angelicin, anti-bacteria, anti-inflammation, osteogenesis

## Abstract

Psoralen and angelicin are two effective compounds isolated from psoraleae, a traditional Chinese medicine. They have a wide range of applications for bone disease treatment and immune modulation. In this study, we explored their new applications for the treatment of periodontal diseases. This study aimed to investigate the effects of psoralen and angelicin on *Porphyromonas gingivalis* growth and *P. gingivalis*-derived lipopolysaccharide (Pg-LPS)-induced inflammation, and further to evaluate their effects on osteogenesis. Finally, the effects of angelicin on a mouse model of periodontitis were also investigated. The results showed that psoralen and angelicin had beneficial dose-dependent effects regarding the inhibition of planktonic *P. gingivalis* and biofilms of *P. gingivalis*. There were no significant differences in the viability of monocyte-like THP-1 cells and human periodontal ligament cells (hPDLCs) treated with either psoralen or angelicin compared to the untreated control cells. Psoralen and angelicin also markedly decreased the mRNA expression and release of inflammatory cytokines (interleukin [IL]-1β and IL-8) by THP-1 cells in a dose-dependent manner. They significantly enhanced the alkaline phosphatase (ALP) activity of hPDLCs and up-regulated the expression of osteogenic proteins (runt-related transcription factor 2 [RUNX2], distal-less homeobox 5 [DLX5], and osteopontin [OPN]). Angelicin significantly attenuated alveolar bone loss and inflammation response in the mice with periodontitis. In conclusion, our data demonstrated that psoralen and angelicin could inhibit the growth of planktonic *P. gingivalis* and *P. gingivalis* biofilm. It is also the first report on the anti-inflammatory effect of psoralen and angelicin against Pg-LPS. They also had an osteogenesis-potentiating effect on hPDLCs. The *in vivo* study also indicated the effect of angelicin regarding protection against periodontitis. Our study highlighted the potential ability of psoralen and angelicin to act as novel natural agents to prevent and treat periodontitis.

## Introduction

Periodontitis is considered to be a kind of chronic infectious disease characterized by periodontal tissue destruction (Slots, 2013). The pathologic features of periodontitis include a high level of inflammatory cell infiltration, destruction of tooth-supporting tissues, and bone loss. Therefore, prevention of periodontitis should inhibit the associated pathogens to control the inflammatory response and guard against more destruction, and treatment should help the periodontal tissues to regenerate (Lu and Shi, 2010). The typical initial therapy for periodontitis involves mechanical methods (such as supragingival scaling, subgingival scaling, and root planing) to clean bacterial plaque (Cao, 2006). Other approaches such as erbium:yttrium-aluminum-garnet (Er:YAG) lasers and toothpastes containing triclosan are also essential treatments. However, complete elimination of the bacteria by these means is impossible because some pathogens can be embedded into the soft tissues. Hence, drug treatment is used as an important adjuvant therapy. This treatment mainly depends on antibiotics, which can result in many problems including drug resistance and oral dysbacteriosis (Palombo, 2011). It has been suggested that for complex chronic disease, treatments that have multiple effects could be more efficacious. Therefore, it is highly necessary to investigate new comprehensive agents that exert anti-bacterial and anti-inflammatory effects and regenerate the damaged periodontal tissues.

Psoraleae is a traditional Chinese medicine and it contains coumarin, terpene phenolic compounds, flavonoids, and so on (Latha et al., 2000). A growing body of research suggests that psoraleae can exert satisfying effects regarding the promotion of bone formation, estrogenic activity, and immune modulation (Lim et al., 2011; Wong and Rabie, 2011). In particular, recent studies have reported that psoraleae inhibits *Staphylococcus aureus* and *Escherichia coli* (Khatune et al., 2004; Kuete et al., 2007; Chopra et al., 2013), and extracts of psoraleae had preventive and protective activities against inflammatory responses (Jin et al., 2014). However, there are no studies on whether these extracts have beneficial effects on periodontal pathogens and *P. gingivalis*-derived lipopolysaccharide (Pg-LPS)-induced inflammation.

**Figure 1:**
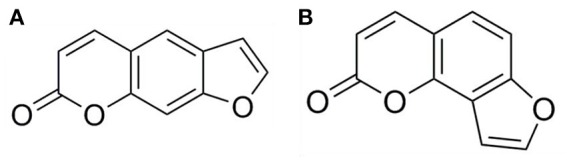
Molecular structure of psoralen **(A)** and angelicin **(B)**.

Psoralen and angelicin are major bioactive compounds of psoraleae, and they are both classified as coumarin compounds (Figure 1). Coumarin is the lactone of 2-hydroxycinnamia acid. Psoralen plus ultraviolet A (UVA) therapy has shown considerable clinical efficacy for the treatment of skin diseases such as psoriasis (Zheng et al., 2010; Heshmati, 2014; Wei et al., 2014). Both psoralen and angelicin have been shown to promote bone formation (Ming et al., 2011; Tang et al., 2011; Li et al., 2014; Xu et al., 2015). Therefore, the present study aimed to evaluate the potential ability of psoralen and angelicin regarding their anti-bacterial and anti-inflammatory effects against *Porphyromonas gingivalis*, a pivotal periodontal pathogen. Their osteogenesis-potentiating effect on human periodontal ligament cells (hPDLCs) was investigated. Their *in vivo* effects on mice with periodontitis were also explored.

## Materials and methods

### Materials, bacterial strains, and growth conditions

Psoralen and angelicin with purity >98% were purchased from Aladdin Chemical Company (Shanghai, China). Drug samples were dissolved in dimethyl sulfoxide (DMSO; Sigma, USA). The standard strain *P. gingivalis* 33,277 was obtained from Shanghai Key Laboratory of Stomatology (Shanghai, China) and grown in tryptic soy broth (TSB; BD, USA) at 37°C anaerobically (80% N_2_; 10% H_2_; 10% CO_2_). The hPDLCs were cultured in Dulbecco's Modified Eagle's medium (DMEM; Hyclone, Logan, UT, USA) with 10% fetal bovine (FBS; Gibco), 100 U/ml penicillin, and 100 μg/ml streptomycin. DMEM Osteogenic Inducing Medium (OIM) contained 2.16 g/L β-glycerophosphate (Sigma), 50 mg/L vitamin C, and 20 μg/L hexadecadrol. Monocyte-like THP-1 cells were cultured in Roswell Park Memorial Institute (RMPI) 1640 Medium and differentiated into macrophage-like cells using 100 ng/ml phorbol 12-mystristate 13-acetate (PMA; Sigma). Both cell types were cultured in a 5% CO_2_/95% air incubator at 37°C.

### Determination of minimum inhibitory concentration (MIC) and minimum bactericidal concentration (MBC)

The MIC and MBC of psoralen and angelicin against planktonic *P. gingivalis* were assessed by two-fold serial dilution methods. Test drugs were prepared at a concentration of 10 mg/ml in DMSO, and 180-μl dilutions of psoralen and angelicin (1.5625–12.5 μg/ml) in TSB were added to a 96-well microplate. In the meantime, a 20-μl aliquot of *P. gingivalis* suspension with a final concentration of 10^7^ CFU/ml was added to a microplate. Control wells containing vehicles co-cultured with or without bacteria were also prepared. The 96-well microplates were incubated under anaerobic conditions for 48 h.

The MIC was defined as the lowest concentration that restrained growth to an optical density at a wavelength of 625 nm of <0.05 (no macroscopically visible growth; Tekbas et al., 2008).

The MBC was determined by plate coating. A 50-μl aliquot of suspension from each well was taken and coated evenly. Bacterial clones were checked after another 5 days of incubation. The MBC was defined as the lowest concentration at which no clone was grown on the agar.

### Biofilm formation assay by crystal violet staining

A crystal violet staining assay was performed to detect the formation of *P. gingivalis* biofilm. Drugs and bacteria were prepared as described, and 200 μl mixed liquor was added into a microplate for 48 h. The bacterial group without drugs was treated as the control. The supernatant of each well was then removed and the wells were washed gently with phosphate-buffered saline (PBS) three times. After that, methanol was used to immobilize the biofilm for 15 min, then 0.04% crystal violet was used for staining for another 15 min. After washing the wells with deionized water, the plate was dried overnight. Lastly, 200 μl ethanol was added to each well for 1 h and the crystalline solid in the bottom was dissolved. The optical density at a wavelength of 550 nm was then recorded. The minimum biofilm inhibition concentration (MBIC_50_) was defined as the lowest drug concentration that resulted in at least 50% inhibition of the formation of biofilm compared with the untreated control (Kong et al., 2015). MBIC_50_ was calculated using Graphpad Prism 6.0, as described previously (Wirjanata et al., 2016).

### Biofilm reduction assay by crystal violet staining

Reduction of *P. gingivalis* biofilm was also detected by a crystal violet staining assay. An equal volume of the *P. gingivalis* suspension at a concentration of 10^7^ CFU/ml was grown in a flat-bottomed 96-well microplate for 48 h to form mature biofilm. After removing the supernatant, PBS was used to gently rinse off the non-adherent cells. TSB medium with drugs of different concentration was then added into the microplate for another 24 h at 37°C anaerobically. The bacterial group without drugs was treated as the control. After that, crystal violet staining was implemented as previously described. The minimum biofilm reduction concentration (MBRC_50_) was defined as the lowest drug concentration that resulted in at least 50% reduction of the biofilm compared with the control (Kong et al., 2015). MBRC_50_ was calculated using Graphpad Prism 6.0, as described previously (Wirjanata et al., 2016).

### Biofilm viability by MTT assay

The effects of psoralen and angelicin on *P. gingivalis* biofilm viability were tested using a 3-(4,5-dimethylthiazolyl-2)-2,5-diphenyltetrazolium bromide (MTT) assay. First, the biofilm was treated in the same way as in the biofilm reduction assay. The medium was extracted and the wells were washed three times with PBS. Then 200 μl MTT (0.5 mg/ml) dilution was added to each well and the microplate was put in the incubator in the dark for 4 h. An equal volume of DMSO was added after extracting the MTT liquid, then the microplate was shaken for 10 min to dissolve the crystals. The absorbance at 490 nm was observed. The sessile MIC (SMIC_50_) was defined as the lowest concentration resulting in at least 50% reduction compared with the untreated control (Kong et al., 2015). SMIC_50_ was calculated using Graphpad Prism 6.0, as described previously (Wirjanata et al., 2016).

### Thickness and viability of *P. gingivalis* biofilm assessed by confocal laser scanning microscopy (CLSM)

A 2-ml aliquot of *P. gingivalis* suspension at a concentration of 10^7^ CFU/ml was added to a 6-well microplate with sterile cover glasses. Drugs at the MIC were added 48 h later. After co-culturing anaerobically with psoralen or angelicin for another 24 h, the cover-glasses were obtained and washed three times with normal saline. The biofilm was stained with a LIVE/DEAD® BacLight™Bacterial Viability Kit (Invitrogen, Carlsbad, USA) for 15 min in the dark and subjected to CLSM to observe the thickness and viability of the *P. gingivalis* biofilm. Typically, the live cells appeared green while the dead cells appeared red in the image.

### Cell culture and cell viability assay

Primary hPDLCs were obtained using a tissue explant method. Premolars extracted for orthodontic reasons were collected from the Oral Maxillo-Facial Surgery Department, Shanghai Ninth People's Hospital, Shanghai Jiao Tong University School of Medicine, China. All patients agreed to participate in the study and signed the informed consent forms. The teeth were then washed at least five times with PBS and the ligament tissues in the middle of the teeth root was scraped. The tissues were placed at the bottom of a Petri dish, and a cover-glass lid was placed on it. Finally, 4 ml DMEM medium with 10% FBS, 100 U/ml penicillin, and 100 μg/ml streptomycin was added. Cells between passage 2 and 5 were used for the experiments. An MTT assay was conducted to evaluate the safety of psoralen and angelicin on hPDLCs. Cells from passage 2 were collected by trypsinization and seeded into a 96-well microplate at a density of 5 × 10^4^/ml. Fresh DMEM with different-concentration drugs was added 24 h later, in place of the original medium. The cells were then grown for another 24 h at 37? in a humidified atmosphere containing 5% CO_2_. At the same time, normal cells without drugs were also prepared as the control. Finally, MTT staining was performed as previously described.

THP-1 cells were cultured in RMPI-1640 with 10% FBS, 100 U/ml penicillin, and 100 μg/ml streptomycin. The medium was changed every 3 days and passaged every week. Cell suspension with 100 ng/ml PMA was grown in a 96-well microplate at a final concentration of 50 × 10^4^/ml. After 24 h, the medium was removed and new medium with drugs was added for another 24 h. Meanwhile, cells without drugs were also prepared as the control. An MTT assay was then performed in the same way.

### Detection of inflammatory and osteogenic gene mRNA expression by real-time (RT)-PCR

Total RNA was extracted as previously described with slight modifications (Ran et al., 2015). The purity and concentration were measured by a spectrophotometer (NanoDrop ND-1000; NanoDrop Technologies, Wilmington, DE, USA). The ratios of the absorbances at wavelengths of 260 and 280 nm were ~1.8–2.0. Reverse transcription was conducted by using the PrimeScript RT reagent kit (Perfect Real Time) (TaKaRa). An RT-PCR assay was performed using SYBR Premix Ex TaqTM II (Perfect Real Time; Takara, Dalian, China) on a Roche LightCycler 480 Real-Time PCR Detection System (Roche, Basel, Switzerland) according to the manufacturer's protocol. Gene expression levels were calculated using the expression 2^−ΔΔ*ct*^; the first Δ means the difference between target and reference gene, and the second Δ means the gap between the experimental and control group. All results were based on at least three independent tests. The genes and primers are listed in Table 1.

**Table 1:** The primer sequence of target genes.

**TARGET GENE**	
GAPDH	Forward: 5′-CGGGAAACTGTGGCGTGAT-3′
	Reverse: 5′-GTCGCTGTTGAAGTCAGAGGAG-3′
IL-1β	Forward: 5′-TGATGGCTTATTACAGTGGC-3′
	Reverse: 5′-TGTAGTGGTGGTCGGAGATT-3′
IL-8	Forward: 5′-ACTCCAAACCTTTCCACC-3′
	Reverse: 5′-CTTCTCCACAACCCTCTG-3′
RUNX2	Forward: 5′-GCGGTGCAAACTTTCTCCAG-3′
	Reverse: 5′-TCACTGCACTGAAGAGGCTG-3′
DLX5	Forward: 5′-GCTCAATCAATTCCCACCTGC-3′
	Reverse: 5′-AGCCCATCTAATAAAGCGTCCC-3′
OPN	Forward: 5′-CCAGCCAAGGACCAACTACA-3′
	Reverse: 5′-AGTGTTTGCTGTAATGCGCC-3′

### Measurement of interleukin (IL)-1β and IL-8 release by enzyme-linked immunosorbent assay (ELISA)

THP-1 cells were seeded in 6-well microplates at a density of 50 × 10^4^/ml. THP-1 cells were pre-treated with psoralen or angelicin for 2 h and then stimulated by 1 μg/ml Pg-LPS for 24 h. The supernatant medium was collected for measurement. After centrifuging, the release of IL-1β and IL-8 in the supernatant was measured by commercial ELISA kits (Neobioscience, Shenzhen, China; Xi'tang Biotechnology, Shanghai, China).

### Osteogenic induction and alkaline phosphatase (ALP) staining of hPDLCs

The hPDLCs were seeded in 12-well microplates at a density of 5 × 10^4^/ml. Cells were divided into four groups: DMEM (the negative control group), osteogenic induction medium (OIM), psoralen, and angelicin group. After 24 h, all cells except the negative control group cells were moved to OIM. Additionally, the psoralen group cells were treated with another 6.25 μg/ml psoralen, and the angelicin group cells contained 3.125 μg/ml angelicin. The medium was changed every 3 days. After inducing osteogenesis for 9 days, ALP staining was performed using an BCIP/NBT Alkaline Phosphatase Color Development Kit (Beyotime, Shanghai, China).

### Osteogenic protein expression assessed by western blot assay

The hPDLCs in three groups were osteogenic inducing for 3 days and lysed by RIPA containing 1% protease inhibitors. Protein concentration was measured by BCA protein assay kit (Beyotime, Shanghai). Equal amounts of protein were separated by SDS-polyacrylamide gel electrophoresis and transferred onto PVDF (Millipore, Billerica, MA, USA) membranes. The membranes were blocked with 5% fat-free milk and incubated overnight with primary antibodies: anti-runt-related transcription factor 2 (RUNX2; 1:500 dilution; Cell Signal Technology, USA), anti-distal-less homeobox 5 (DLX5; 1:500 dilution; Abcam, USA), anti-osteopontin (OPN; 1:500 dilution; Proteintech Group, USA), anti-β-actin (1:2,000 dilution; Santa Cruz Biotechnology). The membrane was washed three times and incubated with secondary antibody (1:5,000 dilution; Santa Cruz Biotechnology) for 1 h. The immunoreaction signals were detected using a chemiluminescence detection kit (Beyotime, Shanghai) and exposed to a gel imaging system.

### Measurement of alveolar bone resorption by micro-computed tomography (CT)

Eight-week-old wild-type male C57BL/6 mice were used for the *in vivo* experiment. The mice were housed at 18–22°C, humidity 55–65%. The mice ate a standard diet and drank randomly. The mice were randomly divided into three groups: control, LPS, and angelicin group. The molar palatal gingival sulcus of the mice in the control group was injected with 6 μl saline three times a week under anesthesia for 4 weeks, while mice in the LPS and angelicin groups were injected with 6 μl Pg-LPS (10 mg/ml, InvivoGen, San Diego, CA, USA). In addition, the mice in the angelicin group were injected with 5 μl angelicin (20 mg/ml) 30 min in advance. The amount of LPS and angelicin was calculated based on earlier research (Wu et al., 2013; Yuan et al., 2016).

Four weeks later, the mice were anesthetized and sacrificed. The maxillae were obtained to detect bone resorption by micro-CT. The maxillae were preserved in paraformaldehyde and then exposed with decalcifying solution, which was replaced every 3 days. The bone structural indices and alveolar bone morphometry were then assessed using vivaCT 40 (SCANCO Medical AG, Zurich, Switzerland). Bone volume percentage (bone volume/total volume, BV/TV), bone surface/bone volume (BS/BV), and bone mineral density (BMD) were calculated with 3-D reconstruction software.

### Anti-inflammation response of angelicin on periodontitis mice assessed by histologic staining assay

The mouse model of periodontitis was the same as previously described. Four weeks later, the mice were anesthetized and sacrificed. The maxillae of mice were obtained to detect the inflammation response by histologic staining assay. The maxillae were fixed by 4% paraformaldehyde and decalcified by 10% EDTA for 1 month. Samples were then dehydrated, embeded and made into sections. Sections were made from mesio-distally direction with a distance of 4 μm. Hematoxylin-eosin (HE) staining assay was used to detect the inflammation response of mice.

### Statistical analysis

All experiments were repeated at least three times. The data are presented as mean ± standard deviation (*SD*). The statistical analysis involved Student's *t*-test and a one-way analysis of variance (ANOVA) followed by a multiple-comparison test. A *P* < 0.05 was considered statistically significant.

## Results

### Effects of psoralen and angelicin on planktonic *P. gingivalis* and *P. gingivalis* biofilm

As shown in Table 2, both psoralen and angelicin exhibited significant anti-bacterial effects against planktonic *P. gingivalis*. The MIC of psoralen and angelicin against *P. gingivalis* was 6.25 and 3.125 μg/ml, respectively. The MBC of each drug was 50 μg/ml.

**Table 2:** Effects of psoralen and angelicin on *P.gingivalis* planktonic and biofilm.

**Drug (μg/ml)**	**Planktonic**	***P.gingivalis***	**Biofilm of** ***P.gingivalis***		
	**MIC**	**MBC**	**Formation**	**Reduction**	**Viability**
			**MBIC_50_**	**MBRC_50_**	**SMIC_50_**
Psoralen	6.25	50	15.8	24.5	5.8
Angelicin	3.125	50	7.5	23.7	6.5

**Figure 2:**
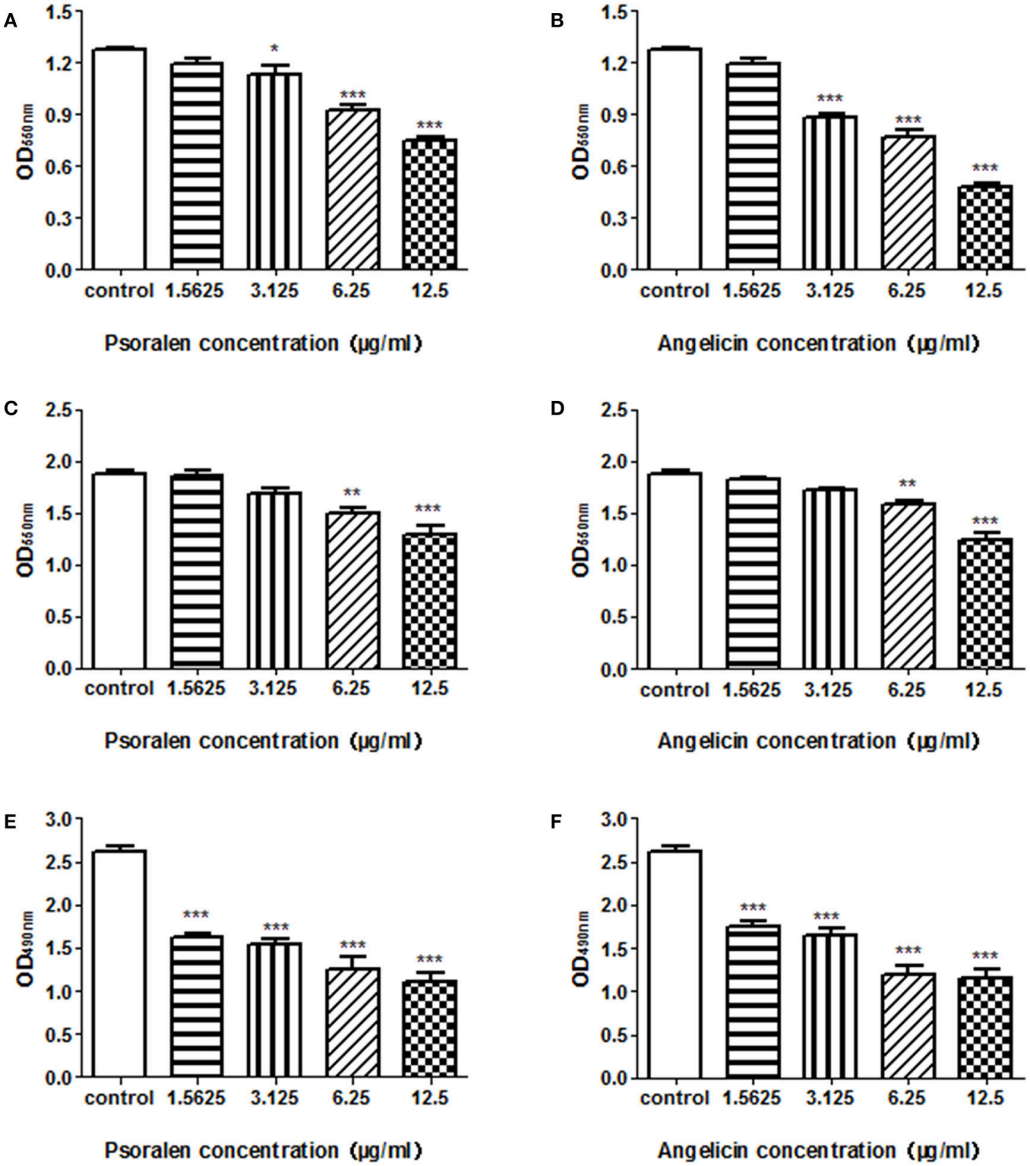
Effects of psoralen and angelicin against *P. gingivalis* biofilm formation, reduction, and viability. *P. gingivalis* was incubated for 48 h in the presence of psoralen **(A)** or angelicin **(B)**. A crystal violet staining assay was conducted and the OD_550nm_ was recorded. Mature biofilm was treated with different concentrations of psoralen **(C)** or angelicin **(D)** for 24 h, and then a crystal violet staining assay was conducted and the OD_550nm_ was recorded. After mature *P. gingivalis* biofilm formed, they were incubated with different concentrations of psoralen **(E)** or angelicin **(F)**. An MTT assay was performed and the OD_490nm_ was observed. Data from three independent experiments are shown as mean ± standard deviation (*SD*). (^*^P < 0.05, ^**^P < 0.01, ^***^P < 0.001 compared with the control group).

To evaluate the effects of psoralen and angelicin on *P. gingivalis* biofilm, crystal violet staining and MTT assays were used to determine the effects on biofilm formation, reduction, and viability. As shown in Figure 2 and Table 2, both psoralen and angelicin inhibited *P. gingivalis* biofilm formation. The MBIC_50_ of psoralen and angelicin was 15.8 and 7.5 μg/ml, respectively. Mature *P. gingivalis* biofilm was eliminated after co-culturing with psoralen or angelicin. Mature biofilm in the psoralen and angelicin groups noticeably decreased over the concentration of 6.25 μg/ml. The MBRC_50_ of psoralen was 24.5 μg/ml, and the MBRC_50_ of angelicin was 23.7 μg/ml. In addition, psoralen and angelicin could also inhibit the viability of *P. gingivalis* biofilm. The two compounds decreased the viability of mature *P. gingivalis* biofilm in a dose-dependent manner. The SMIC_50_ of psoralen and angelicin was 5.8 and 6.5 μg/ml, respectively. All these data indicated that psoralen and angelicin had beneficial effects regarding the inhibition of planktonic *P. gingivalis* as well as biofilms.

### Effects of psoralen and angelicin on *P. gingivalis* biofilm, as assessed by CLSM

**Figure 3:**
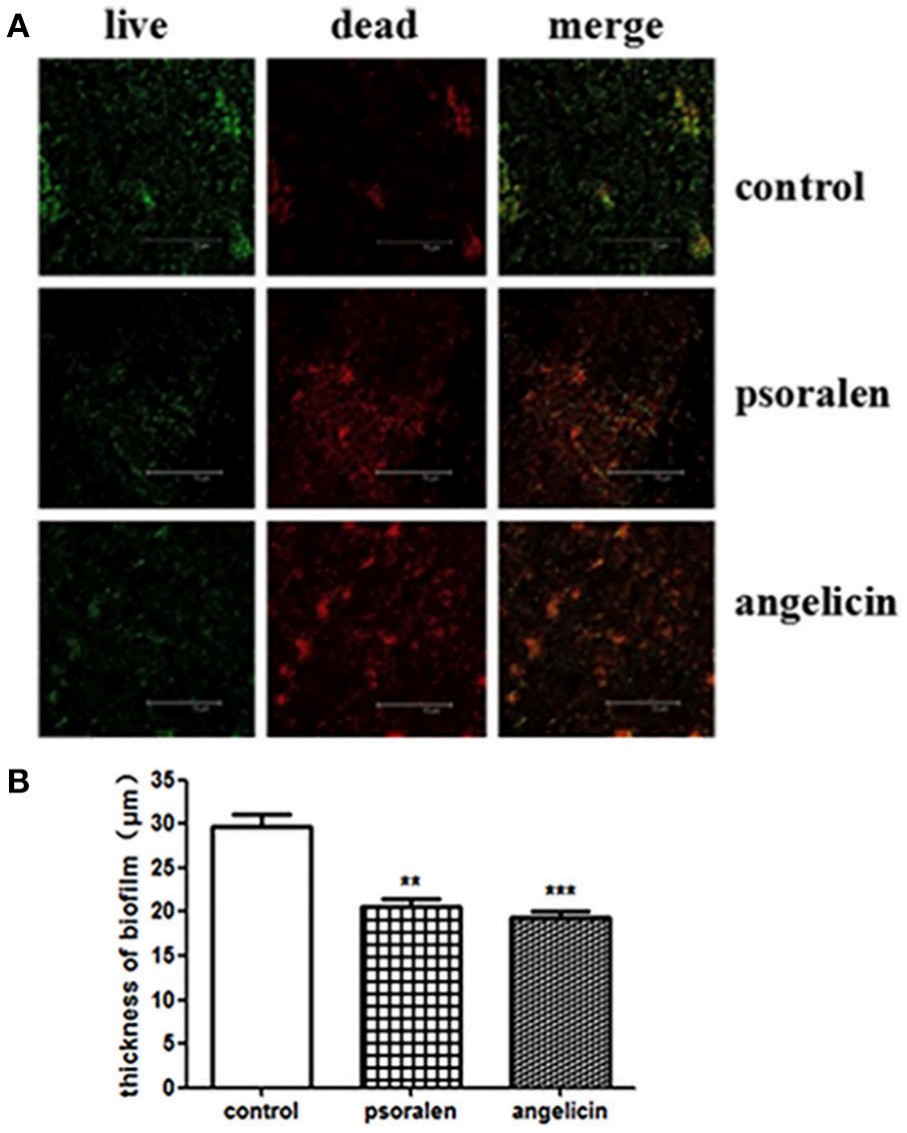
Viability and thickness of *P. gingivalis* biofilm, as assessed by CLSM**. (A)** CLSM images of *P. gingivalis* biofilm in the presence of psoralen or angelicin. Cells were stained using a LIVE/DEAD Bacteria Viability Kit and images were captured by CLSM. Live cells were stained green whereas dead bacteria were stained red. **(B)** Effects of psoralen and angelicin on *P. gingivalis* biofilm thickness. Scale bar = 75 μm. Data are shown as mean ± standard deviation (*SD*). (^**^P < 0.01, ^***^P < 0.001 compared with the control group).

The thickness and viability of mature biofilm were investigated by CLSM. As shown in Figure 3, when treated with 6.25 μg/ml psoralen or 3.125 μg/ml angelicin, the biofilm viability markedly decreased compared with that in the control group. In addition, the thickness of biofilm in the drug-treated groups was much lower than that in the control group. In the control group, the biofilm thickness was about 30 μm, but the value dropped down to ~21 and 19 μm in the psoralen and angelicin groups, respectively. These results suggested that psoralen and angelicin had inhibitory effects on *P. gingivalis* biofilm.

### Effects of psoralen and angelicin on viability of THP-1 cells and hPDLCs

**Figure 4:**
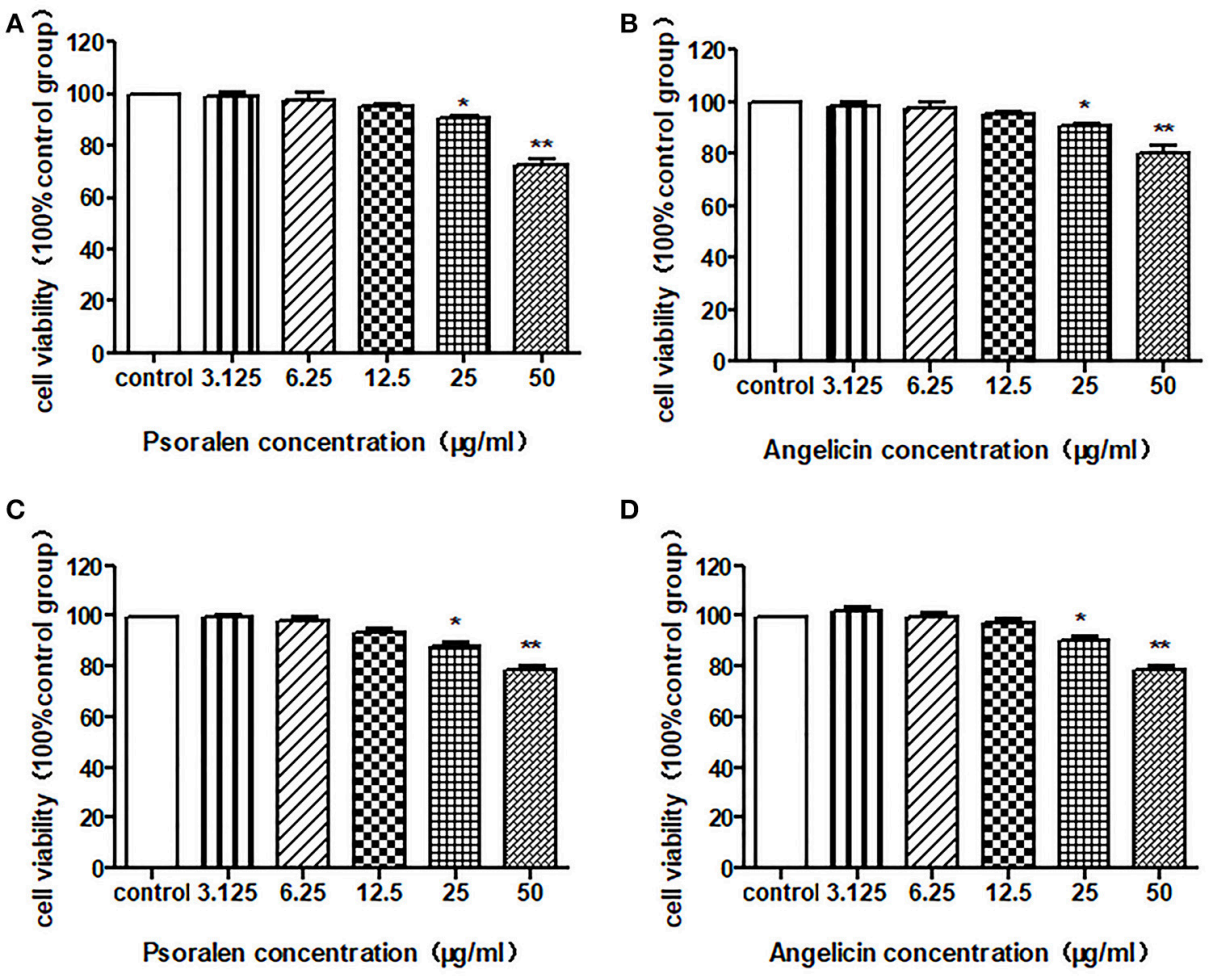
Cytotoxic effect of psoralen and angelicin on hPDLCs and THP-1 cells**. (A)** Cytotoxic effect of psoralen on hPDLCs. **(B)** Cytotoxic effect of angelicin on hPDLCs. **(C)** Cytotoxic effect of psoralen on THP-1 cells. **(D)** Cytotoxic effect of angelicin on THP-1 cells. Cells were treated with different concentrations of drugs for 24 h and an MTT assay was used to detect the cell viability. Data from three independent experiments are shown as mean ± standard deviation (*SD*). (^*^P < 0.05, ^**^P < 0.01 compared with the control group).

An MTT assay was conducted to detect the cell viability after treatment with psoralen or angelicin. As shown in Figure 4, neither psoralen nor angelicin had a cytotoxic effect on THP-1 cells at concentrations below 25 μg/ml. Similarly, there was no difference between the groups with concentrations below 25 μg/ml and the control group on the hPDLCs.

### Inhibition of Pg-LPS-induced inflammatory response in THP-1 cells by psoralen and angelicin

**Figure 5:**
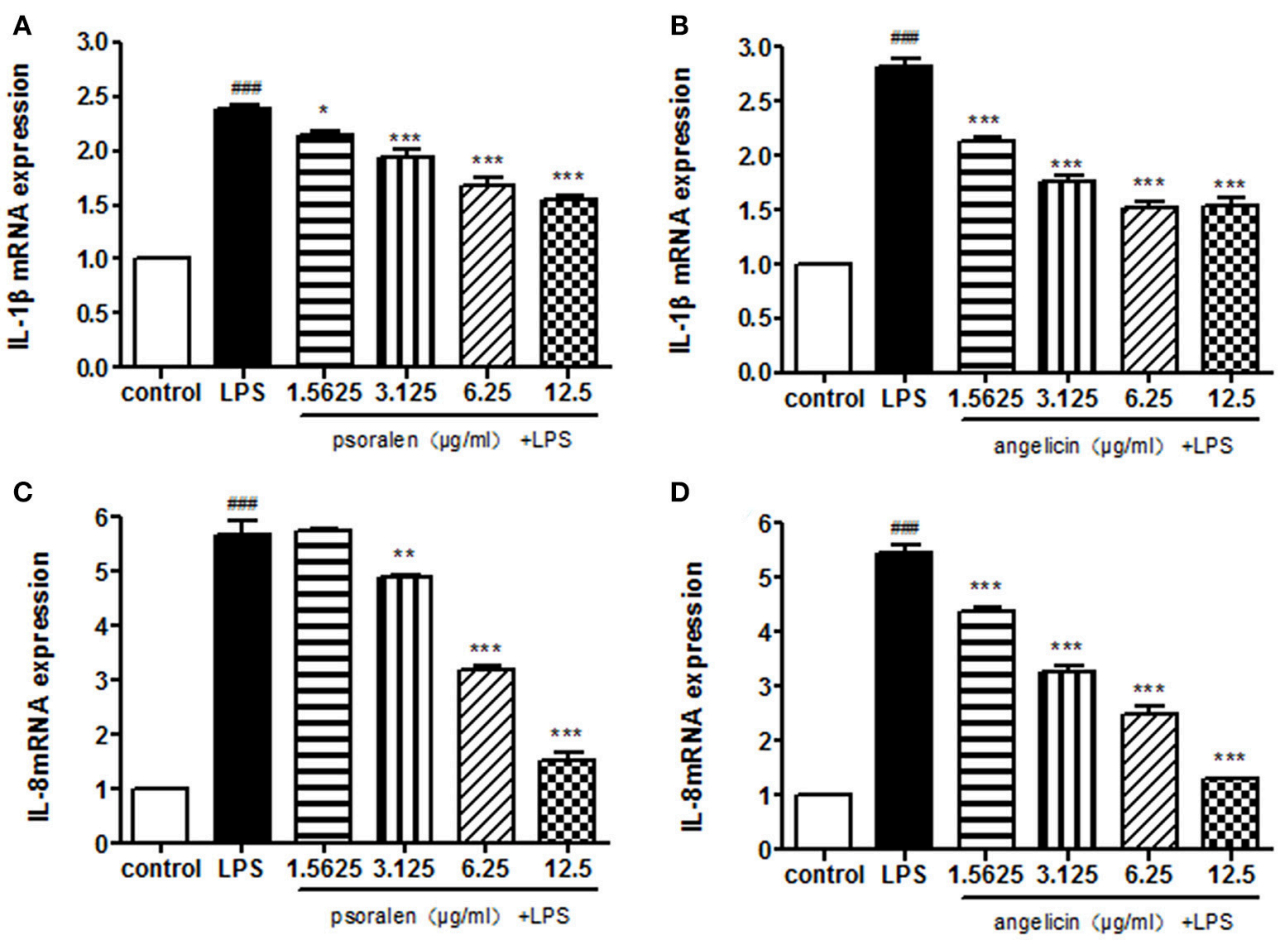
Effects of psoralen and angelicin on mRNA expression of IL-1β and IL-8, as assessed by RT-PCR. **(A)** Effects of psoralen on IL-1β mRNA expression. **(B)** Effects of angelicin on IL-1β mRNA expression. **(C)** Effects of psoralen on IL-8 mRNA expression. **(D)** Effects of angelicin on IL-8 mRNA expression. THP-1 cells were pre-treated with psoralen and angelicin for 2 h and then stimulated with 1 μg/ml Pg-LPS for 4 h. RT-PCR was used to determine the IL-1β and IL-8 mRNA expression levels. Data from three independent experiments are shown as mean ± standard deviation (*SD*). (^*^P < 0.05, ^**^P < 0.01, ^***^P < 0.001 compared with the LPS group. ^###^*P* < 0.001 compared with the control group).

The expression of IL-1β and IL-8 was measured quantitatively by RT-PCR. As shown in Figure 5, psoralen significantly inhibited LPS-induced IL-1β and IL-8 mRNA expression compared with the expression levels in the LPS group. Angelicin also prevented the increase of IL-1β and IL-8 mRNA expression mediated by LPS.

**Figure 6:**
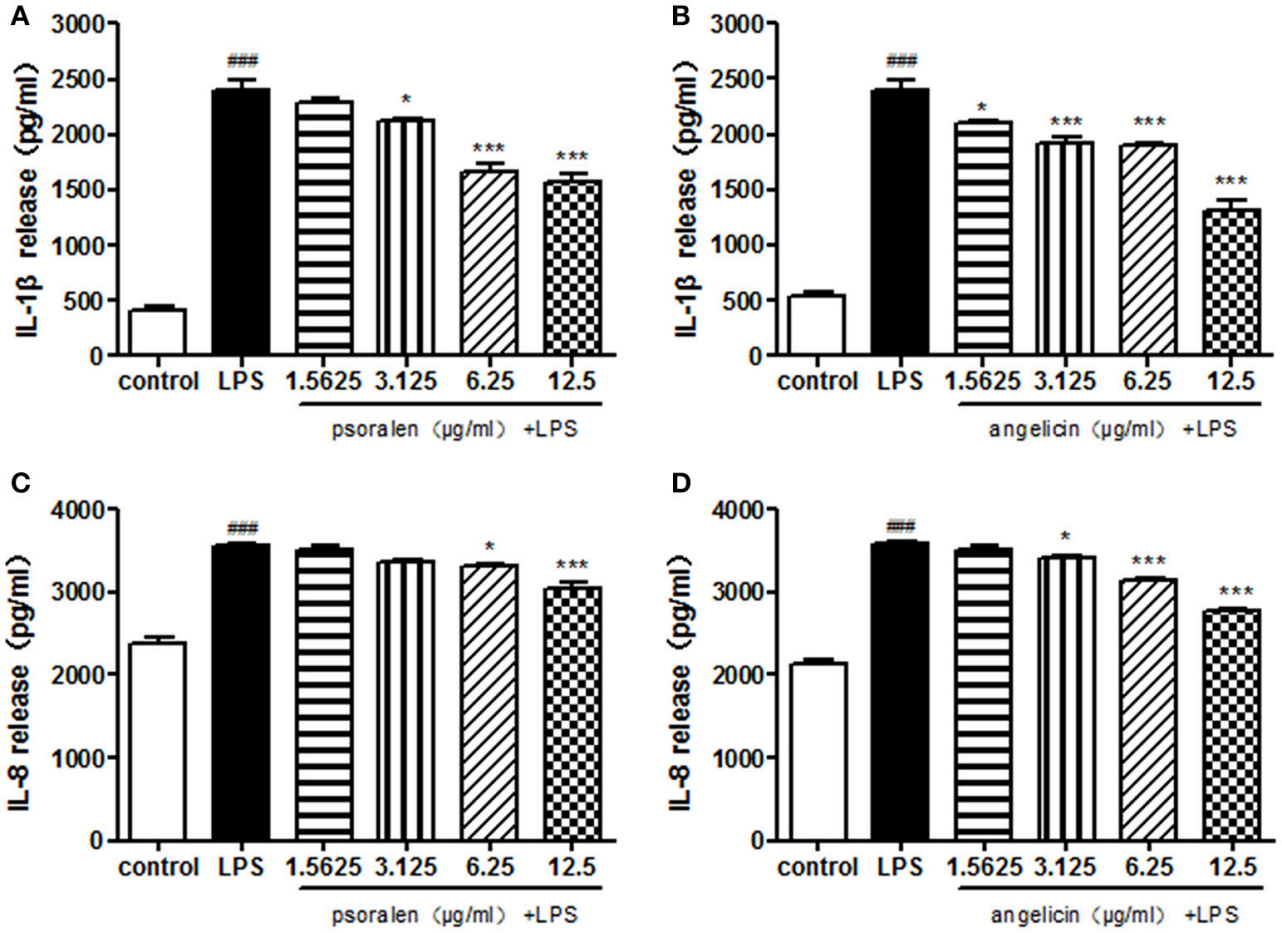
Effects of psoralen and angelicin on IL-1β and IL-8 release, as assessed by ELISA. **(A)** Effects of psoralen on IL-1β release. **(B)** Effects of angelicin on IL-1β release. **(C)** Effects of psoralen on IL-8 release. **(D)** Effects of angelicin on IL-8 release. We have made corresponding change in the manuscript. THP-1 cells were pre-treated with psoralen and angelicin for 2 h and then stimulated with 1 μg/ml Pg-LPS for 24 h. ELISA was used to evaluate the cytokine release. Data from three independent experiments are shown as mean ± standard deviation (*SD*). (^*^P < 0.05, ^***^P < 0.001 compared with the LPS group. ^###^*P* < 0.001 compared with the control group).

Cytokine release was determined by ELISA. As presented in Figure 6, Pg-LPS significantly increased IL-1β and IL-8 secretion to ~2,500 and 3,500 pg/ml, respectively. Pre-treatment with psoralen significantly decreased the release of IL-1β and IL-8 in a dose-dependent manner. Similarly, angelicin also inhibited the IL-1β and IL-8 release in a dose-dependent manner. The results suggested that psoralen and angelicin could attenuate the inflammatory response induced by LPS.

### Psoralen and angelicin increased ALP activity, as assessed by ALP staining

**Figure 7:**
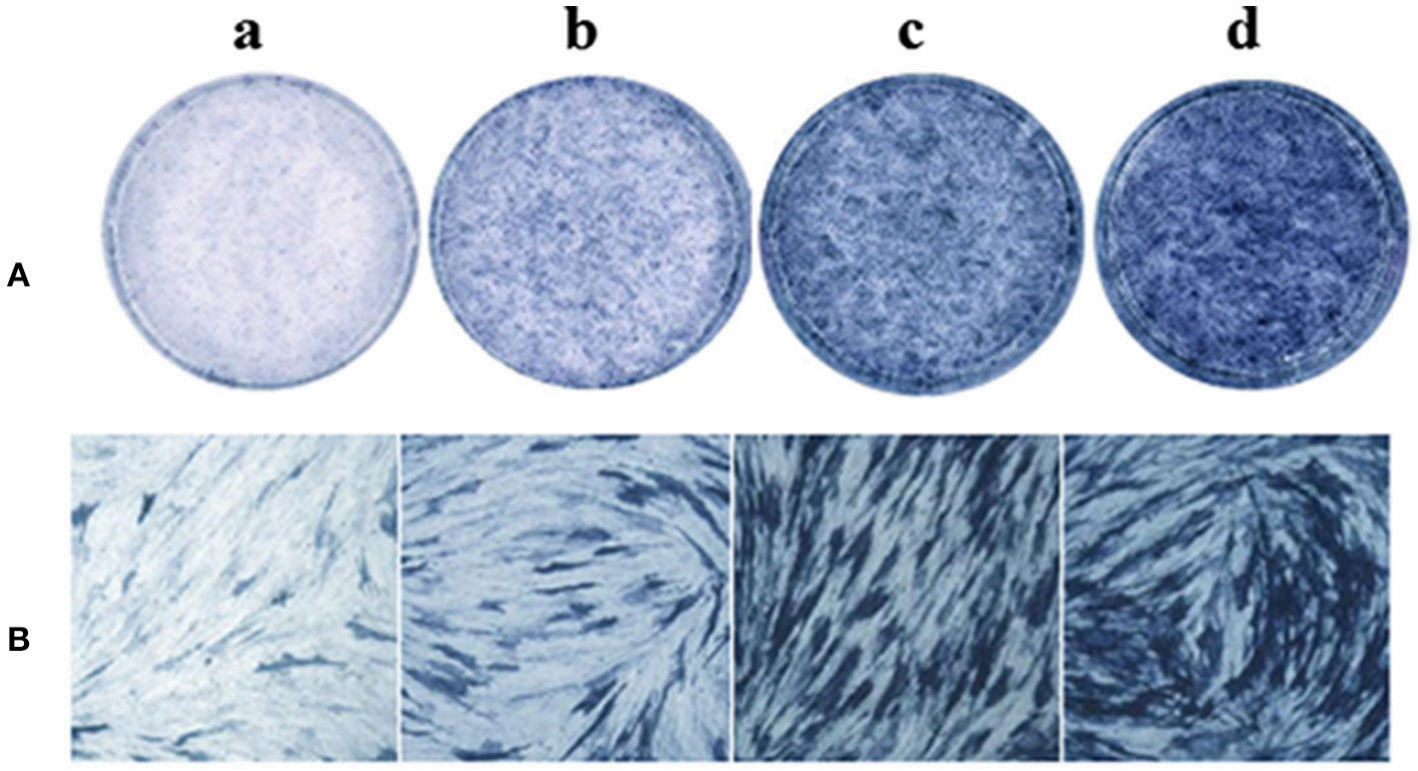
Effects of psoralen and angelicin on ALP activity of hPDLCs. The hPDLCs were subjected to osteogenic induction for 9 days and ALP staining was tested using a one-step BCIP/NBT kit. (a) DMEM group (b) osteogenic inducing medium (OIM) group (c) psoralen group (d) angelicin group. **(A)** Images were captured by scanning. **(B)** Microscope images at a magnification of 100×.

The effect of psoralen and angelicin on osteogenic differentiation of hPDLCs was evaluated by ALP activity. After cells were induced for 9 days, ALP staining was performed using an BCIP/NBT Alkaline Phosphatase Color Development Kit. As presented in Figure 7, cells in the psoralen group and the angelicin group were obviously stained deeper than the OIM group. The results highlighted the osteogenesis effects of psoralen and angelicin on hPDLCs.

### Psoralen and angelicin enhanced osteogenic gene expression and protein expression of hPDLCs

**Figure 8:**
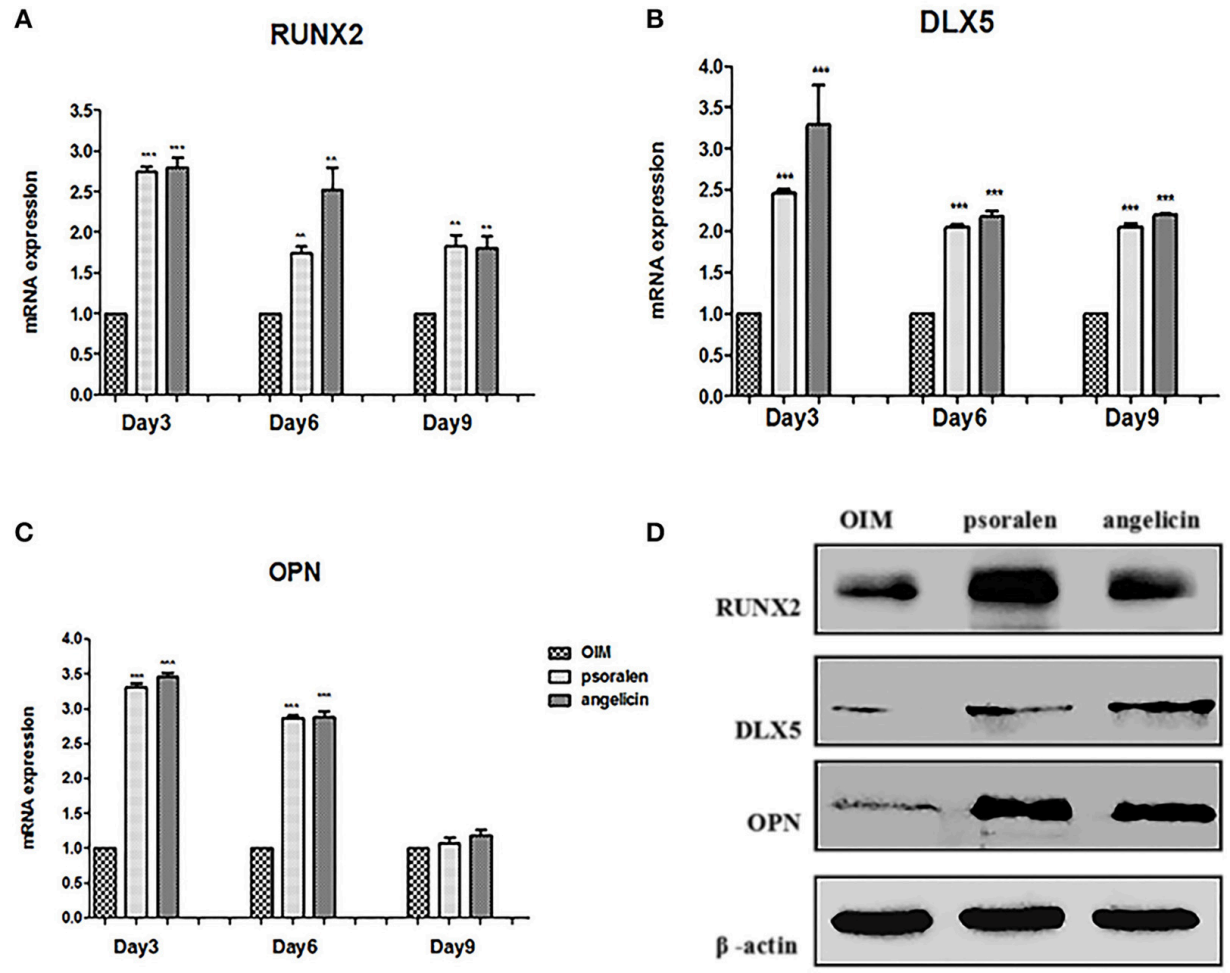
Effects of psoralen and angelicin on osteogenic genes and proteins of hPDLCs. **(A)** mRNA expression of RUNX2 in hPDLCs. **(B)** mRNA expression of DLX5 in hPDLCs. **(C)** mRNA expression of OPN in hPDLCs. Sets of hPDLCs were induced for 3, 6, and 9 days, respectively. RT-PCR was used to determine osteogenic gene mRNA expression. Data from three independent experiments are shown as mean ± standard deviation (*SD*). (^*^P < 0.05, ^**^P < 0.01, ^***^P < 0.001 compared with the OIM group). **(D)** protein expression of RUNX2, DLX5, and OPN on hPDLCs. The hPDLCs were subjected to osteogenic induction for 3 days and western blot was used to determine RUNX2, DLX5, and OPN protein expression.

The mRNA expression of osteogenic genes was measured by RT-PCR. As presented in Figure 8, when treated with psoralen and angelicin, mRNA expression of RUNX2 and DLX5 were clearly enhanced compared to the expression in the OIM group at days 3, 6, and 9. In addition, mRNA expression of OPN was also elevated at days 3 and 6.

Osteogenic protein expression was measured by western blot at day 3. As shown in Figure 8, protein expression of RUNX2, DLX5, and OPN was clearly enhanced compared to the expression in the OIM group, which was similar to the RT-PCR results. Together, these data indicated that psoralen and angelicin had significant osteogenesis-potentiating effects on hPDLCs.

### Angelicin prevented the alveolar bone resorption and inflammation response in mice with periodontitis

**Figure 9:**
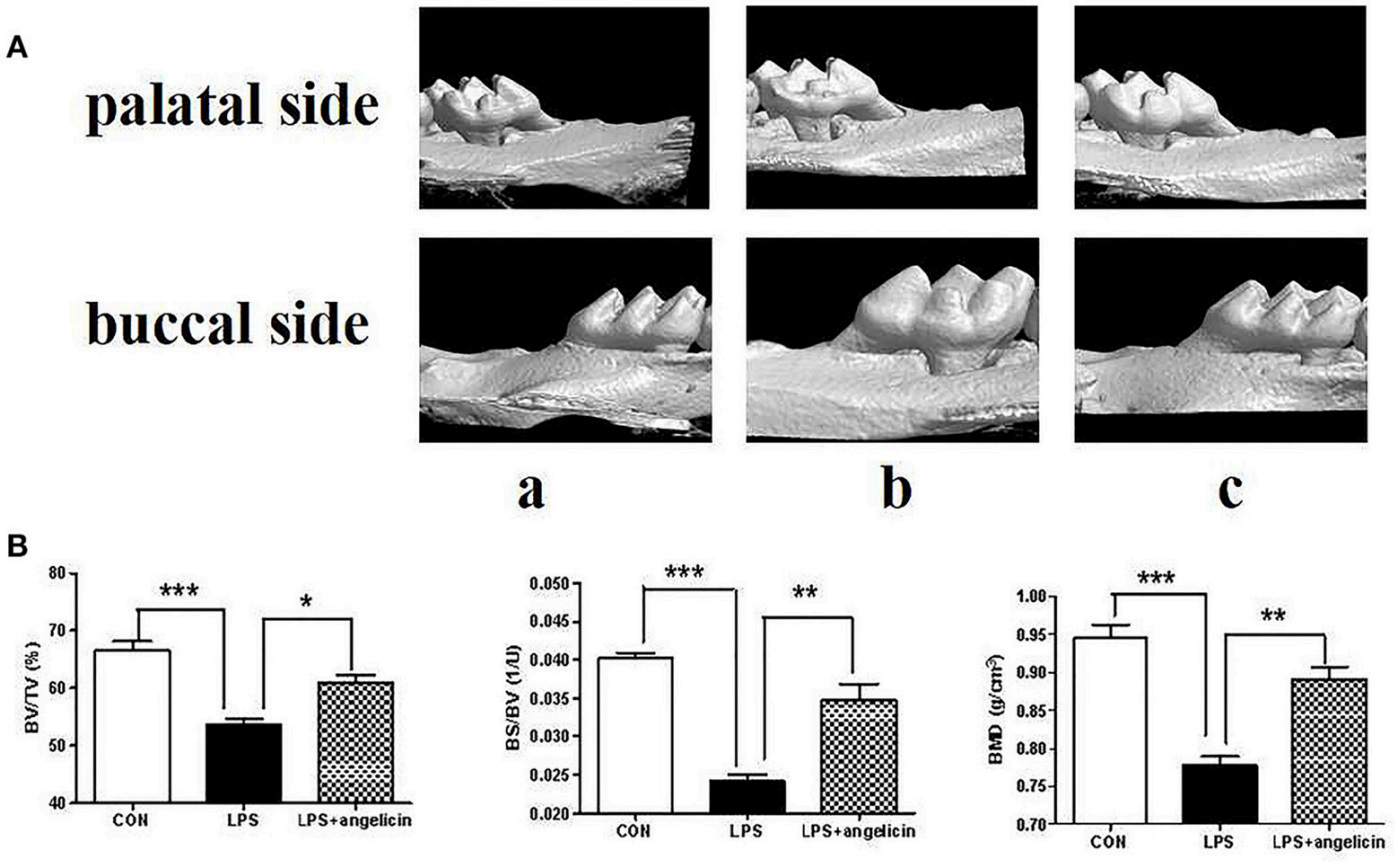
Effects of angelicin on alveolar bone in mice with periodontitis. **(A)** Images captured by micro-CT scanning of molar region. (a) Control group (b) LPS group (c) LPS+angelicin group. **(B)** Bone density analysis of alveolar bone. Data from three independent experiments are shown as mean ± standard deviation (*SD*). (^*^P < 0.05, ^**^P < 0.01, ^***^P < 0.001 compared with the LPS group).

Alveolar bone resorption was investigated by micro-CT. As shown in Figure 9, there was a significant difference between the control and LPS groups. Alveolar bone loss was observed in the mice treated with Pg-LPS. However, in the angelicin group, the posterior maxillary bone loss was reduced and close to level in the control group. The micro-CT method provided high-resolution results for analyzing the bone micro-structure. Relative bone volume and alveolar thickness were also analyzed. As shown in Figure 9B, the BV/TV, BS/BV, and BMD in the LPS group were markedly decreased compared with those in the control group, while pre-treatment with angelicin significantly attenuated the alveolar bone loss. Taken together, the results showed that the posterior maxillary bones of mice with periodontitis were obviously decreased compared with the bones of the mice in the control group, which was significantly alleviated by angelicin.

**Figure 10:**
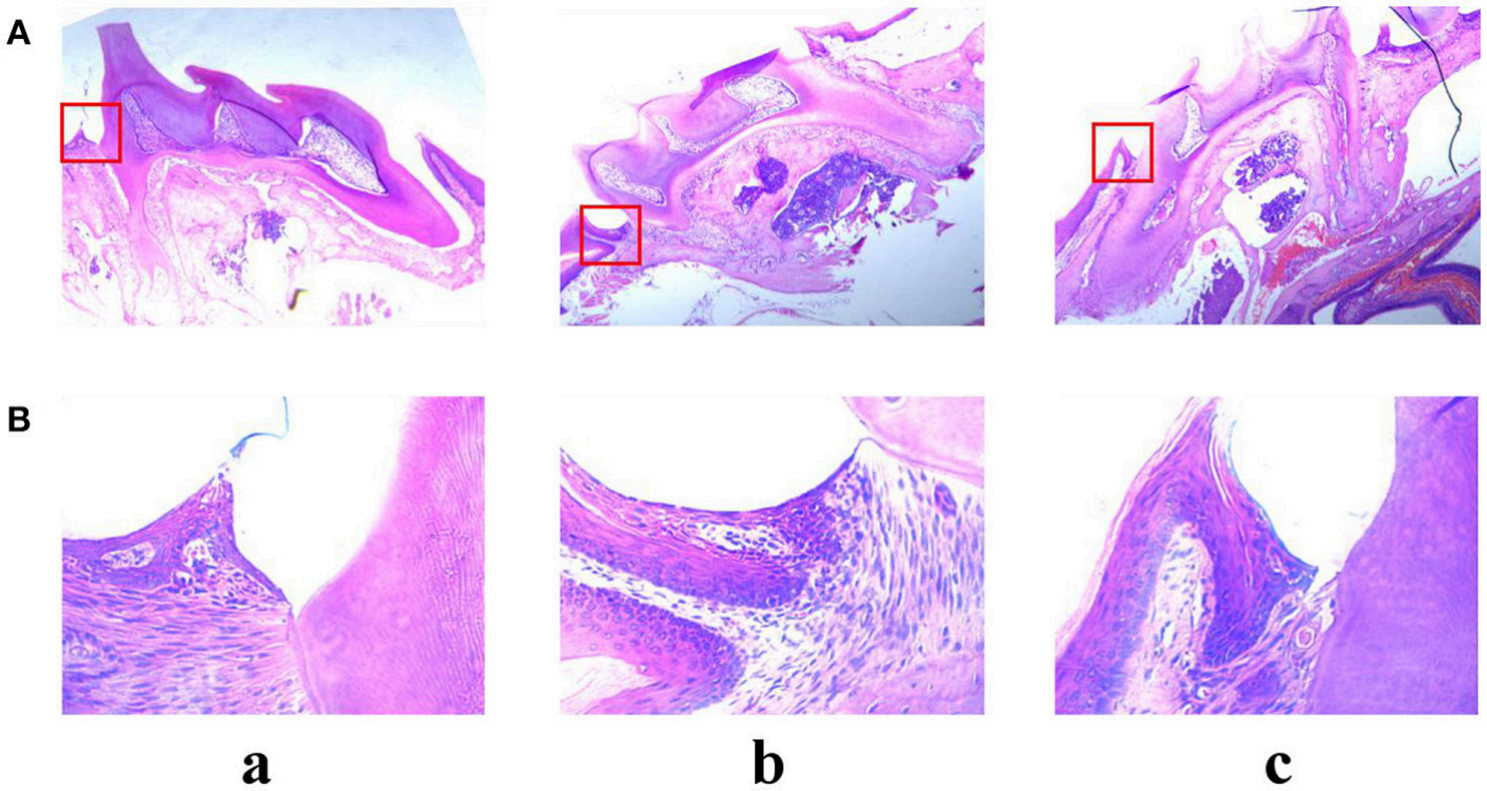
Effects of angelicin on inflammation response in mice with periodontitis. The maxillae of mice were obtained to detect the inflammation response by hematoxylin-eosin staining assay. (a) Control group (b) LPS group (c) LPS+angelicin group. Microscope images were at a magnification of 50× **(A)** and 400× **(B)**.

Alveolar bone inflammation response was detected by hematoxylin-eosin staining assay. As shown in Figure 10, there was a significant difference between control group and LPS group. In the LPS group, many neutrophil cells were observed in the connective tissue compared with the control group. However, pre-treatment with angelicin (Figure 10c) significantly attenuated the inflammatory cell infiltration in the gingival tissue. The results showed that angelicin could reduce the inflammation response induced by LPS in mice.

## Discussion

Periodontitis is a chronic inflammatory disease that results in periodontal tissue destruction, and the investigation of new comprehensive treatment agents is urgently needed. Psoralen and angelicin are two effective compounds of Psoraleae, and they have long been used in bone formation and skin diseases, such as osteomalacia, osteoporosis and lichen planus (Chopra et al., 2013; Alsenaid et al., 2016). As the saying goes, “old drugs, new tricks.” In this study, we explored their new applications in the treatment of periodontitis. This study highlighted the anti-bacterial effects of psoralen and angelicin on planktonic *P. gingivalis* and *P. gingivalis* biofilm. It is also the first report on the anti-inflammatory effect of psoralen and angelicin against Pg-LPS. Their effects on periodontal bone formation were also investigated, and the results suggest that psoralen and angelicin might be novel effective agents for periodontal treatment.

It is well-known that the initial factor involved in periodontal diseases is dental plaque biofilm, which contains many microorganisms (Liu et al., 2013). Among the pathogenic bacteria, *P. gingivalis*, a Gram-negative anaerobic bacterium, plays a key role in periodontitis, especially in chronic periodontitis (Wakabayashi et al., 2010). It has been reported that psoraleae It has been reported that psoraleae exhibited anti-microbial effects on several microorganisms, such as *S. aureus, Streptococcus* spp., and so on (Yin et al., 2004; Wanqchuk et al., 2014; Cui et al., 2015). It could also affect the membrane permeability of *Staphylococcus aureus* and change the content of extracellular soluble protein (Zhou, 2016). Psoraleae was also effective against adherent cells of *S. mutans* in water-insoluble glucan (Chopra et al., 2013). However, there are no reports on the impact of psoraleae on oral pathogens, particularly on periodontal pathogens. In our study, we examined the effects of psoralen and angelicin on a key periodontal pathogen, *P. gingivalis*. Both drugs not only inhibited the growth of planktonic *P. gingivalis* but also impaired *P. gingivalis* biofilm by inhibiting biofilm formation, eliminating established biofilm, and reducing biofilm viability. However, the underlying mechanism of the anti-bacterial effects is uncertain. One possibility we deduced is that the two coumarin compounds could disrupt the cell membrane, causing cell splitting and death. Both psoralen and angelicin are liposoluble, so they can get though the plasma membrane and destroy the integrity of the cytomembrane (Yin et al., 2004).

Antigen components and other virulence factors produced by bacteria could directly impair the periodontal tissues. They can also induce the host immune-inflammatory response, which can cause further damage to periodontal tissues (Feng and Weinberg, 2006; Alex and Richard, 2011). The accumulation of inflammatory cells induced by virulence factors is considered one of the most important pathogenic factors in periodontitis. Localized infections activate tissue-resident cells, which include dendritic cells, macrophages, and so on. During the process, various immune cells, such as monocytes, neutrophils, and activated T cells, infiltrate from the blood into the gingival sulcus. These activated cells can produce and release inflammatory mediators, such as ILs, tumor necrosis factors (TNFs), and so on (Ryu et al., 2016; Zhou et al., 2016). These cytokines, in turn, augment the chemotactic signals that are generated during inflammatory responses. The cascade amplification response stimulates immune cells to synthesize and secrete a wide variety of inflammatory products, which cause further tissue damage. IL-1β, which is usually produced by activated macrophages, is a significant mediator of the inflammatory response. An *in vitro* study proved that gingival fibroblasts from periodontitis patients expressed more IL-1β compared with the gingival fibroblasts from healthy subjects (Baek et al., 2013). Similarly, the IL-8 level in gingival crevicular fluid was significantly associated with the periodontal situation (Lagdive et al., 2013). Psoralen was reported to treat arthritis by regulating the balance of Th1/Th2 cells and inhibiting the expression of TNF-α, IL-6, and IL-1β (Zhang et al., 2017). It was also reported that psoralen and angelicin could treat osteoarthritis due to the estrogen-like effects (Tang, 2017). In our research, IL-1β and IL-8 expression was found to be significantly increased after stimulation with LPS. However, after treatment with psoralen and angelicin, their levels were down-regulated. Therefore, our research indicated that Pg-LPS can induce an inflammatory response in THP-1 cells. This response was clearly attenuated by psoralen and angelicin.

RUNX2 is a key transcription factor associated with osteoblast differentiation. DLX5 is a protein that plays an important role in bone development and healing. Current research indicates that DLX5 is important in appendage development. OPN is usually secreted by osteoblasts, osteocytes, and osteoclasts. It is involved in the mineralization and absorption process of the bone matrix. We found that psoralen and angelicin promoted osteogenic differentiation by elevating ALP activity and up-regulated osteogenic genes and proteins (RUNX2, DLX5, and OPN). The osteogenesis ability of psoralen and angelicin on periodontal ligament cells was in accordance with the results of many other studies (Ming et al., 2011; Xu et al., 2015). Psoralen and angelicin were reported to be able to ameliorate sex hormone deficiency-induced osteoporosis in mice (Yuan et al., 2016). It is known that IL-1β and IL-8 has a close relationship with osteoclasts and could result in bone loss (Gamonal et al., 2001; Baek et al., 2013). In our experiment, psoralen and angelicin significantly decreased the expression of IL-1β and IL-8, correspondingly, they could lead to a reduction in bone loss. In addition, their underlying mechanism of action has been reported to be related to their estrogen-like effects (Yuan et al., 2016). We also propose that their estrogen-like effects could explain their osteogenesis effects on hPDLCs. However, further studies are still needed to investigate how psoralen and angelicin promote bone formation in periodontal tissues.

In conclusion, our experiments demonstrated that psoralen and angelicin had significant anti-bacterial effects on planktonic bacteria and bacterial biofilm. This is also the first report on the anti-inflammatory effect of psoralen and angelicin against Pg-LPS. Psoralen and angelicin could promote osteogenesis in periodontal tissue without cytotoxicity. Angelicin also attenuated alveolar bone loss and inflammation response in mice with periodontitis, providing protection against periodontitis. Taken together, these new application of psoralen and angelicin could be harnessed to treat and prevent periodontitis.

## Ethics statement

All experimental protocols were approved by the ethical committee of the Animal Care and Experimental Committee of Shanghai Jiao Tong University School of Medicine.

## Author contributions

ZS and WZ design the experiments. XL, CY, YH, XX, and YL executed the experiments. JZ, HC, and WL collected all data. XL analyzed the data and wrote the manuscript. ZS and WZ made critical revision. All authors gave final approval and are accountable for the work.

### Conflict of interest statement

The authors declare that the research was conducted in the absence of any commercial or financial relationships that could be construed as a potential conflict of interest.
